# Short and Long-Term Outcomes of Lesion Index-Guided High-Power Short-Duration Approach for Atrial Fibrillation Ablation

**DOI:** 10.3390/jcm12154986

**Published:** 2023-07-28

**Authors:** Andrea Di Cori, Matteo Parollo, Francesco Gentile, Lorenzo Pistelli, Carlo Vitale, Salvatore Della Volpe, Mario Giannotti Santoro, Lorenzo Mazzocchetti, Raffaele De Lucia, Antonio Canu, Valentina Barletta, Gino Grifoni, Luca Segreti, Maria Grazia Bongiorni, Giulio Zucchelli

**Affiliations:** 1 Second Division of Cardiology, Cardiac-Toracic and Vascular Department, University Hospital of Pisa, 56124 Pisa, Italy; a.dicori@gmail.com (A.D.C.); frage1993@gmail.com (F.G.); pis.lorenzo@gmail.com (L.P.); im.carlo.vitale@gmail.com (C.V.); dellavolpesalvatore86@gmail.com (S.D.V.); gsmario88@yahoo.it (M.G.S.); lorenzomazzocchetti90@gmail.com (L.M.); r.delucia.md@gmail.com (R.D.L.); a.canu25@gmail.com (A.C.); valentina.barletta@ao-pisa.toscana.it (V.B.); ginogrifoni@gmail.com (G.G.); l.segreti@ao-pisa.toscana.it (L.S.); bongiorni.maria@gmail.com (M.G.B.); zucchelli76@gmail.com (G.Z.); 2 Cardiology Unit, Department of Clinical and Experimental Medicine, University Hospital “G. Martino”, University of Messina, 98168 Messina, Italy

**Keywords:** atrial fibrillation, atrial fibrillation ablation, high-power short-duration, lesion index, pulmonary vein isolation

## Abstract

High-power short-duration (HPSD) ablation is an increasingly used ablation strategy for pulmonary vein isolation (PVI) procedures, but Lesion Index (LSI)-guided HPSD radiofrequency (RF) applications have not been described in this clinical setting. We evaluated the procedural efficiency and safety of an LSI-guided HPSD strategy for atrial fibrillation (AF) ablation. Paroxysmal and persistent AF patients scheduled for AF ablation were prospectively enrolled and divided into two groups, according to the ablation power used (≥45 W for the LSI-HP Group and ≤40 W for the LSI-LP group). All patients underwent only PVI LSI-guided ablation (5.5 to 6 anteriorly; 5 to 5.5 superiorly, 4.5 to 5 posteriorly) with a point-by-point strategy and an inter-lesion distance <6 mm. Forty-six patients with AF (25 in the LSI-HP Group vs 21 in the LSI-LP Group)—59% paroxysmal, 78% male, with low-intermediate CHA_2_DS_2_-Vasc scores (2 [1–3]), a preserved ejection fraction (65 ± 6%) and a mean left atrial index volume of 39 ± 13 mL/m^2^ were prospectively enrolled. Baseline clinical characteristics were comparable between groups. PVI was successful in all patients. The RF time (29 (23–37) vs. 49 (41–53) min, *p* < 0.001), total procedure time (131 (126–145) vs. 155 (139–203) min, *p* = 0.007) and fluoroscopy time (12 (10–18) vs. 21 (16–26) min, *p* = 0.001) were significantly lower in the LSI-HP Group. No complications or steam pops were seen in either group. LSI-HP AF ablation significantly improved procedural efficiency—reducing ablation time, total procedural duration, and fluoroscopy use, while maintaining a comparable safety profile to lower-power procedures.

## 1. Introduction

Atrial fibrillation (AF) is the most common cardiac arrhythmia in adults worldwide and is linked to significant morbidity and mortality, becoming over time a primary concern in terms of public health and healthcare expenditure [1]. Over the years, catheter ablation (CA) has emerged as a highly effective, safe, and dependable option for achieving rhythm control in patients suffering from either paroxysmal or persistent AF [2,3,4]. Radiofrequency (RF) pulmonary vein isolation (PVI) serves as the foundational technique for AF ablation. Its effectiveness depends on the creation of transmural, continuous, and enduring lesions that can guarantee the persistence of PVI over time. On the other hand, the aim of a durable PVI should be counterbalanced by the risk of overshooting, keeping in mind the principle “primum non nocere”. 

The Lesion Index (LSI; Abbott, Abbott Park, IL, USA) is a proprietary algorithm developed to help operators in balancing the effectiveness and safety of catheter ablation. Contact force, RF application duration, and RF current are all considered together in a weighted formula and expressed in a number [5], providing to the operator real-time feedback regarding lesion formation during energy delivery.

In recent years, “high power short duration” (HPSD) techniques have garnered attention for their potential to reduce procedural times [6,7]. These methods involve the delivery of a higher wattage—up to 50 W—for brief periods to generate faster, yet equally effective, lesions. Nevertheless, this strategy may be associated with an increased risk of steam pops or overheating because of the higher power employed. Thus, it appears to have become even more relevant to identify tools capable of preventing high-power side-effects. In this context, LSI may play a central role in ensuring the safety of the HPSD procedure; however, it has not yet been validated in this context.

In this study, we aim to evaluate an LSI-guided HPSD strategy in comparison to a standard low-power LSI-guided approach, focusing on their efficacy, safety, and efficiency. By doing so, we aim to determine whether an LSI-guided HPSD approach can offer improved outcomes for patients with atrial fibrillation, while also minimizing the potential risks associated with the procedure.

## 2. Materials and Methods

### 2.1. Patient Population

Patients referred to our Centre for AF ablation (PVI only) using Abbott EnSite^TM^ mapping suite from March 2019 to September 2019 were prospectively enrolled. The inclusion criteria were:

- Documented paroxysmal or persistent AF (<12 months) with an indication for PVI-only RF ablation;
- Aged 18 years or older;
- Able to sign an informed consent form and willing to compete the required study procedures during follow-up.

According to the ablation strategy, enrolled subjects were concurrently non-randomly allocated into two groups as per the operator’s choice:

- HPSD group: patients who underwent PVI with an LSI-guided HPSD strategy (≥45 W);
- Standard group: patients who underwent PVI with LSI-guided standard PVI (≤40 W).

Exclusion criteria are available in the Supplementary Materials.

The Declaration of Helsinki was adequately addressed, and the study was approved by the local ethics committee.

### 2.2. Ablation Procedure

**Figure 1:**
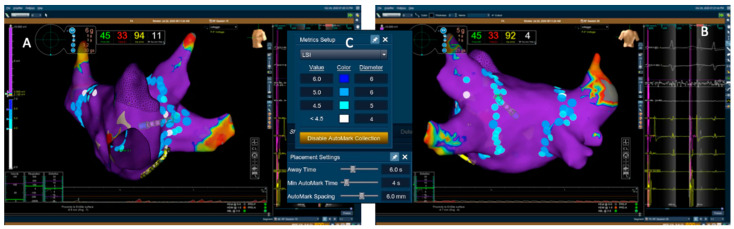
Electroanatomical map of LA and PV after continuous circular RF application. (**A**). Left Anterior Oblique view. (**B**). Postero-anterior view. (**C**). The color of the Automarks are coded according to the LSI target. The color of the Automarks are coded according to the LSI target. PVI, pulmonary vein isolation; LA, left atrium; LSI, ablation index; PV, pulmonary vein; RF, radiofrequency.

All procedures were performed using the EnSite^TM^ Precision (Abbott, Abbott Park, IL, USA) electro-anatomical mapping suite under conscious sedation. Procedures were performed, irrespective of the ablation strategy used, by the same experienced operators with more than 10 years of experience in atrial fibrillation ablation. Femoral vein access was obtained using ultrasound guidance; transseptal puncture was performed using a steerable sheath under fluoroscopic guidance. A multipolar high density mapping catheter (Advisor HD Grid, Abbott, Abbott Park, IL, USA) was used to obtain an anatomical and voltage map of the left atrium (LA) and subsequently exchanged with a contact force-enabled RF ablation catheter (TactiCath SE, Abbott, Abbott Park, IL, USA). Wide antral circumferential ablation (WACA) was performed with a point-to-point technique for both the left and right pulmonary veins, with the inclusion of the carina as per the operator’s choice. A maximum 6 mm interlesion distance was maintained. Both for the HPSD and Standard group, the LSI targets were 5.5–6 for the anterior wall, 5–5.5 for the superior wall, and 4.5–5 for the posterior wall (Figure 1). PVI was confirmed by documenting bidirectional block during CS pacing and pacing from inside the vein with the ablation catheter.

### 2.3. Follow-Up

Follow-up was performed at 3, 6, and 12 months after the ablation procedure. Extended follow-up was also performed in May 2022. All patients underwent clinical examination, 12-lead ECG, and 24 h Holter at all follow-up visits. All antiarrhythmic drugs (AADs) were to be stopped at the 3-month follow-up visit if no other indication for AAD therapy was met. Successful ablation was defined as an absence of documented atrial tachycardia (AT), AF, or atrial flutter (AFL) in the 12-lead ECG or 24 h Holter, as well as the absence of clinical symptoms highly suggestive of AF recurrence. Recurrences occurring during the first 90 days after ablation were not considered for efficacy assessment (“blanking period”).

### 2.4. Statistical Analysis

Continuous variables are expressed as mean ± standard deviation in case of normal distribution or median (25°–75° interquartile range) in case of non-normal distribution. Categorial variables are express as *n* (%). Categorial variables were compared using Pearson’s Chi square test. Continuous variables of normal distribution were compared using Student’s T test; in case of non-normal distribution, the Mann–Whitney test was performed. Overall ablation efficacy was analyzed using the Log rank test and Kaplan–Meier curves; 2-tailed *p* values less than 0.05 were considered to be significant. All statistical analysis was performed with SPSS 26 software (IBM, Armonk, NY USA).

## 3. Results

### 3.1. Study Population

Forty-six patients (mean age 65 ± 7 years, 78% men) were enrolled, referred to our center for radiofrequency ablation of either paroxysmal (*n* = 27, 59%) or persistent AF (*n* = 19, 41%). Hypertension was the most common risk factor (63%), followed by smoking (44%), dyslipidemia (26%), and diabetes mellitus (13%). Only two patients (4%) had a previous history of ischemic heart disease and/or of heart failure, and the median CHA_2_DS_2_VASc score was two (1–3). At echocardiography, patients showed good systolic function (mean left ventricular ejection fraction 65 ± 6%) and a slightly enlarged left atrium (mean indexed volume 39 ± 13 mL/m^2^). At the time of recruitment, 32 (69%) patients were on antiarrhythmics drugs (mainly of class IC, 52%) and most patients were taking a direct oral anticoagulant (94%).

As detailed in Table 1, no significant differences in the baseline characteristics emerged when comparing patients assigned to the HPSD procedure (*n* = 25) to those undergoing a standard power ablation procedure (*n* = 21; all *p* > 0.05).

### 3.2. Ablation Procedure

All patients underwent pulmonary vein isolation during the index procedure, with a similar first-pass success rate between the two groups (48% vs. 44%). No significant differences emerged when comparing the necessity of right carina ablation, while a better trend was observed for the left carina in the HPSD Group. Similarly, no differences were documented for sinus rhythm restoration through either radiofrequency delivery or external electric synchronized cardioversion (all *p* > 0.05).

**Figure 2:**
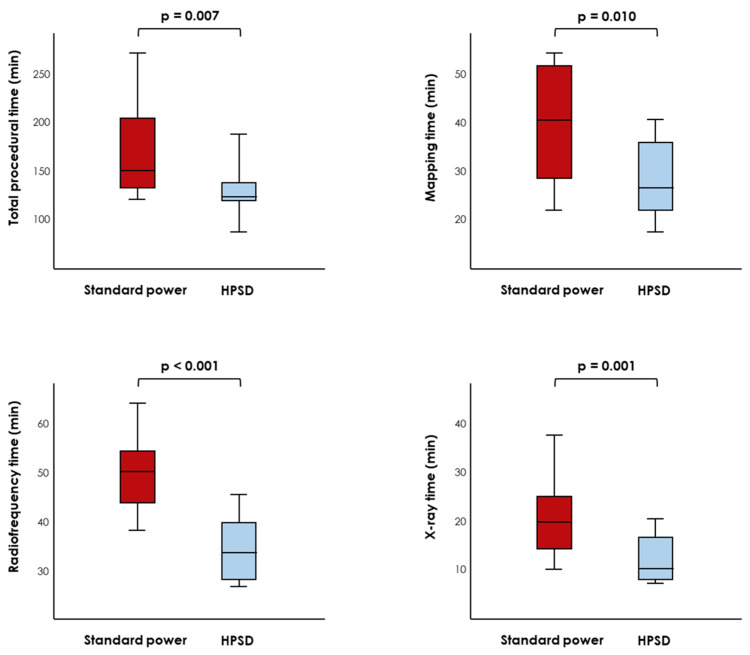
Procedural times in patients undergoing a high-power short-duration (HPSD) vs. a traditional ablation procedure.

When comparing procedural times, significant differences were observed between the groups. Compared with a traditional procedure, the use of an HPSD technology was associated with a significantly shorter procedure (131 (126–145) vs. 155 (139–203) min, *p* = 0.007), secondary to a reduction in both mapping (25 (20–35) vs. 40 (27–52) min, *p* = 0.010) and radiofrequency (29 (23–37) vs. 49 (41–53) min, *p* < 0.001) times. Additionally, the time of exposure to fluoroscopy was significantly shortened in the HPSD group (12 (10–18) vs. 21 (16–26) min, *p* = 0.001; Table 2 and Figure 2).

No major periprocedural complications were reported. As for non-major complications, a single case of access site vascular complication was reported in the non-HPSD group and two cases of pericardial effusion not requiring drainage were reported (one for each group; Table 3).

### 3.3. Clinical Follow-Up

**Figure 3:**
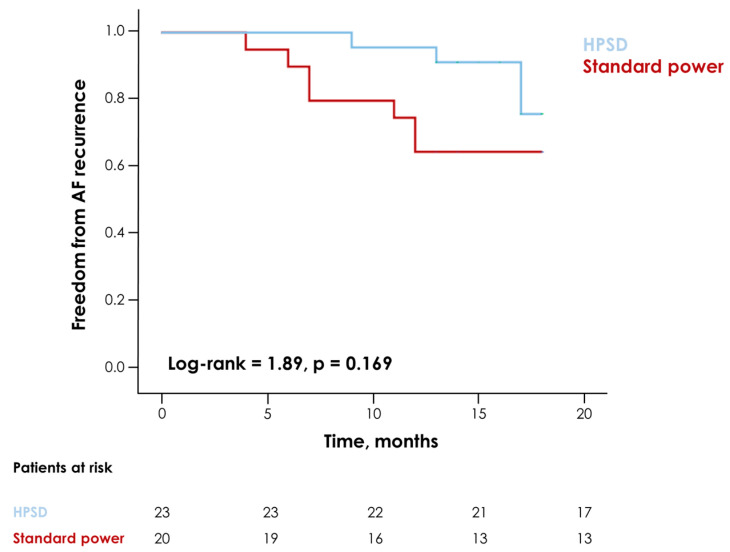
Kaplan–Meier curves for the risk of atrial fibrillation (AF) recurrence during follow-up in patients undergoing a high-power short-duration (HPSD) vs. a traditional ablation procedure.

**Figure 4:**
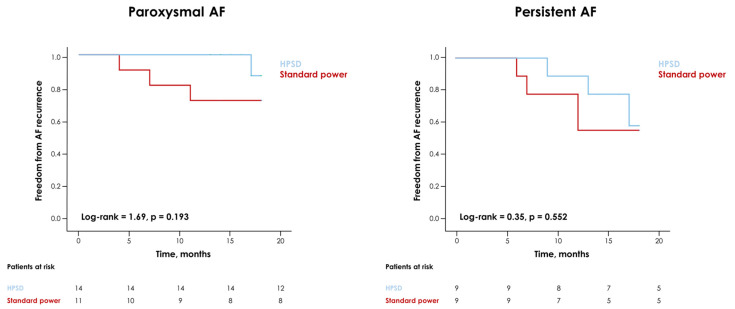
Kaplan–Meier curves for the risk of atrial fibrillation (AF) recurrence during follow-up in patients undergoing a high-power short-duration (HPSD) vs. a traditional ablation procedure with either paroxysmal or persistent AF.

During a median follow-up of 17 (13–18) months, 11 (26%) cases of AF recurrence were reported, with no statistically significant differences between the two groups (Figure 3). Similar findings were observed when considering patients with either paroxysmal or persistent AF separately (Figure 4).

No differences were observed either for medical treatments during follow-up or for the necessity of either external electric synchronized cardioversion or a redo ablation procedure (all >0.05).

## 4. Discussion

The most significant findings of our study highlighted that a pulmonary vein isolation (PVI) Lesion Index (LSI)-guided high-power short-duration (HPSD) strategy:

- Significantly enhanced procedural efficiency, resulting in reduced overall procedural and fluoroscopic time;
- Achieved comparable acute and long-term efficacy of PVI;
- Demonstrated a similar level of procedural safety.

As aforementioned, the decrease in procedural time was primarily attributable to the substantial reduction in radiofrequency (RF) delivery due to the high-power strategy. The use of a high power allows for shorter RF times at each site, mitigating common problems such as catheter instability due to heart motion and patient breathing. However, both acute and long-term efficacy and safety were found to be comparable to the standard approach, further reinforcing the reliability of HPSD approaches in obtaining durable lesions.

AF ablation aims to determine transmural lesions, which can last well over time, ensuring permanent vein isolation. The main factors influencing this outcome include the power delivery, RF time, catheter tip force on the target tissue, and catheter stability [8,9,10]. The advent of contact force-sensing catheters has provided a better and direct definition of the tip–tissue relationship, while improvements in mapping system technology has allowed for higher precision and catheter stability even during RF delivery. The need for multiple parameters in one index has been addressed by the introduction of the Ablation Index (Biosense Webster, Diamond Bar, CA, USA) and Lesion Index (Abbott, Abbott Park, IL, USA). Notwithstanding the fact that it was developed before the widespread adoption of HPSD techniques, the Ablation Index has already been validated in such contexts in terms of acute and long-term procedural efficacy and safety in both medium- and small-scale studies [11]. On the other hand, data regarding LSI are extremely limited [12], and—at this point—limited to the context of low-power approaches.

Furthermore, despite the evident efficiency of HPSD, energy titration and ablation targets have not yet been clearly defined, but are still based on individual decisions—with potential implications for both safety and lesion durability. Impedance drops, ICE surveillance, and electrogram changes are sometimes used as surrogates for lesion effectiveness, but in most cases, the only domain which defines RF delivery is time (not longer than 8–10 s). Furthermore, the safety-to-efficacy window for HPSD appears to be narrow, often requiring additional tools to prevent tissue overheating and steam pop formation.

Our study is the first to evaluate the safety and effectiveness of LSI as a metric system to support not only the validated low-power long-duration (LPLD) strategy, but also an HPSD one. Our data confirmed that LSI is a valid and reliable tool even in the context of HPSD procedures. In the acute setting, no differences were observed in the first-pass isolation rate, with a non-significant, yet interesting trend towards a lower need for ablation of the left carina in the HPSD Group. These results seem to suggest that LSI-guided high-power RF application reduced the residual conduction gap after circular RF application around the PV antrum and inter-venous carina, compared with the conventional group. This is consistent with ex vivo animal models, where the lesion diameter has been reported to be significantly larger, and the lesion depth significantly smaller, with increasing RF power. The effectiveness and durability of LSI-guided lesions, with respect to an inter-lesion distance < 6 mm, was clearly confirmed by a comparable freedom from AF recurrences during follow-up.

At the beginning of the HPSD era, safety was a major concern for most operators—particularly regarding tissue overheating and steam pops. Chen et al. assessed esophageal lesion rates when performing PVI with an Ablation Index-powered HPSD, revealing a markedly low incidence of endoscopically detected esophageal injury [13]. In our experience, neither steam pops nor major complications (including clinically overt esophageal fistulae or perforation) were observed at the LSI target values used in both groups. However, given the small population, the study was not sufficiently powered to assess such rare complications. Metrics estimating the overall amount of energy delivered at any single point seem to work independently from the RF delivery strategy used.

In future, improvements in procedural imaging [14] or in catheter technology (such as the implementation of high-density mapping catheters [15], ablation catheters with multiple surface thermocouples, or the utilization of new energy sources such as pulsed field ablation), in combination with advanced non-invasive hemodynamic monitoring [14] will allow the prevention and reduction of procedural-related complications. These improvements could potentially optimize the balance between safety and efficacy, further enhancing the reliability of HPSD techniques in various clinical settings.

## 5. Conclusions

In conclusion, our study demonstrated that an LSI-guided HPSD strategy can consistently improve procedural efficiency while maintaining comparable acute and long-term efficacy and safety, as the standard low-power approach. The LSI metric system proved to be a valid and reliable tool, even within the context of HPSD procedures, offering promise for the continued development and refinement of HPSD strategies in the field of atrial fibrillation ablation. Further studies are needed to confirm these results.

## 6. Study Limitations

Our study has several limitations: First of all, this is a non-randomized study with no blinding for operators and patients to the chosen ablation strategy. The overall population was small relative to the long-term freedom from AF and rare adverse event assessment. HPSD or standard strategy choice was up to the individual electrophysiologist. Follow-up was conducted with clinical monitoring, 12-lead ECG, and 24-Holter for relapse identification, while no continuous invasive or non-invasive rhythm monitoring was performed; therefore, subclinical relapses may have been underdiagnosed. In terms of safety assessment, as already pointed out in the discussion, no endoscopic esophageal examinations were routinely performed—posing a risk for subclinical damage underdiagnosis.

## Figures and Tables

**Table 1:** Baseline features of the study population and comparisons between patients undergoing a high-power short-duration (HPSD) vs. a traditional ablation procedure.

	All Patients (*n* = 46)	Controls (*n* = 21)	HPSD (*n* = 25)	*p*
**Clinical features**				
Age, years	65 ± 7	65 ± 9	64 ± 6	0.624
Males, *n* (%)	36 (78)	19 (91)	17 (68)	0.084
Hypertension, *n* (%)	29 (63)	14 (67)	15 (60)	0.762
History of smoking, *n* (%)	20 (44)	10 (48)	10 (40)	0.321
Diabetes, *n* (%)	6 (13)	3 (14)	3 (12)	1.000
Dyslipidemia, *n* (%)	12 (26)	7 (33)	5 (20)	0.335
OSAS, *n* (%)	1 (2)	1 (5)	0 (0)	0.457
IHD, *n* (%)	2 (4)	1 (5)	1 (4)	1.000
History of HF, *n* (%)	2 (4)	0 (0)	2 (8)	0.493
CHA2DS2VASc score	2 (1–3)	2 (1–2)	2 (1–3)	0.576
Symptomatic AF, *n* (%)	42 (91)	20 (95)	22 (88)	0.881
Paroxysmal AF, *n* (%)	27 (59)	12 (57)	15 (60)	1.000
History of atrial flutter, *n* (%)	8 (17)	6 (29)	2 (8)	0.117
History of AVNRT, *n* (%)	3 (7)	0 (0)	3 (12)	0.239
**Echocardiography**				
LA diameter, mm	42 ± 6	42 ± 5	42 ± 6	0.757
LA area, cm^2^	24 ± 7	23 ± 4	25 ± 8	0.373
LAVi, ml/m^2^	39 ± 13	39 ± 13	40 ± 13	0.662
LVEF, %	65 ± 6	58 ± 5	61 ± 7	0.282
**Medical therapy**				
Class IC AADs, *n* (%)	24 (52)	13 (62)	11 (44)	0.253
Class III AADs, *n* (%)	6 (13)	8 (38)	14 (56)	0.253
Oral anticoagulant, *n* (%)	43 (94)	20 (95)	23 (92)	1.000

Values are mean ± SD, median (interquartile range), or *n* (%). AAD: anti-arrhythmic drugs; AF: atrial fibrillation; AVNRT: atrioventricular nodal reentrant tachycardia; HF: heart failure; IHD: ischemic heart disease; LA: left atrium; LAVi: left atrium volume index; LVEF: left ventricular ejection fraction; OSAS: obstructive sleep apnea syndrome.

**Table 2:** Technical and temporal details of the index ablation procedure in patients undergoing a high-power short-duration (HPSD) vs. a traditional ablation procedure.

	All Patients (*n* = 46)	Controls (*n* = 21)	HPSD (*n* = 25)	*p*
**Procedural details**				
First pass, *n* (%)	21 (46)	10 (48)	11 (44)	1.000
Left carina ablation, *n* (%)	13 (28)	9 (43)	4 (16)	0.056
Right carina ablation, *n* (%)	11 (24)	6 (28)	5 (20)	0.730
Ablation AF-resolution, *n* (%)	4 (9)	3 (14)	1 (4)	0.318
ECV AF-resolution, *n* (%)	24 (48)	11 (53)	13 (52)	0.883
**Times**				
Procedural time, hours	2.3 (2.1–3.1)	2.6 (2.3–3.4)	2.2 (2.1–2.4)	**0.007**
Procedural time, minutes	140 (128–185)	155 (139–203)	131 (126–145)	**0.007**
Mapping time, minutes	37 (28–48)	40 (27–52)	25 (20–35)	**0.010**
Radiofrequency time, minutes	37 (28–48)	49 (41–53)	29 (23–37)	**<0.001**
X-ray time, minutes	17 (11–22)	21 (16–26)	12 (10–18)	**0.001**

Values are *n* (%) or median (IQR). AF: atrial fibrillation; ECV: external electric cardioversion.

**Table 3:** Procedural complications and longitudinal data.

	All Patients (*n* = 46)	Controls (*n* = 21)	HPSD (*n* = 25)	*p*
**Periprocedural complications**				
Vascular complications, *n* (%)	1 (2)	1 (5)	0 (0)	0.457
Pericardial effusion, *n* (%)	2 (4)	1 (5)	1 (4)	1.000
Cardiac tamponade, *n* (%)	0 (0)	0 (0)	0 (0)	1.000
Stroke/TIA, *n* (%)	0 (0)	0 (0)	0 (0)	1.000
Tracheoesophageal fistula, *n* (%)	0 (0)	0 (0)	0 (0)	1.000
Phrenic nerve lesion, *n* (%)	0 (0)	0 (0)	0 (0)	1.000
PV stenosis, *n* (%)	0 (0)	0 (0)	0 (0)	1.000
**Longitudinal data**				
Recurrence, *n* (%)	11 (26)	7 (35)	4 (17)	0.295
Recurrence time, months	17 (13–18)	18 (12–18)	17 (14–18)	0.990
ECV at follow-up, *n* (%)	4 (9)	2 (10)	2 (9)	1.000
Redo at follow-up, *n* (%)	2 (5)	1 (5)	1 (4)	1.000
Class IC AADs at follow-up, *n* (%)	32 (70)	15 (71)	17 (68)	1.000
Class III AADs at follow-up, *n* (%)	12 (26)	6 (29)	6 (24)	0.749
Rate control at follow-up, *n* (%)	16 (37)	9 (45)	7 (30)	0.361
Anticoagulants at follow-up, *n* (%)	30 (70)	15 (75)	15 (65)	0.526

Values are *n* (%) or median (IQR). HPSD: high-power short-duration PV: pulmonary vein; TIA: transitory ischemic attack, ECV: external electric cardioversion, AAD: anti-arrhythmic drugs.

## Data Availability

The data presented in this study are available on request from the corresponding author. The data are not publicly available due to privacy reasons.

## Supplementary Materials

The following supporting information can be downloaded at: https://www.mdpi.com/article/10.3390/jcm12154986/s1, Study exclusion criteria.
