# Supplementary material for: Short and Long-Term Outcomes of Lesion Index-Guided High-Power Short-Duration Approach for Atrial Fibrillation Ablation
Source: J Clin Med. 2023 Jul 28;12(15):4986. doi: 10.3390/jcm12154986 (PMC10420312; doi:10.3390/jcm12154986)
Supplement: Supplementary file 1 [file jcm-12-04986-s001.zip › jcm-2478849-supplementary.pdf]

SUPPLEMENTAL MATERIAL

Study exclusion criteria

- Long-standing persistent atrial fibrillation defined as continuous AF greater than 12 months in duration.
- Previous ablation or surgery in the left atrium.
- Implanted left atrial appendage occlude.
- Implanted mitral or tricuspid valve replacement.
- Implanted cardiac defibrillator (ICD).
- Participation in another clinical investigation that may confound the results of this study.
- Pregnant or nursing.
- Presence of other anatomic or comorbid conditions, or other medical, social, or psychological conditions that, in the investigator's opinion, could limit the subject's ability to participate in the clinical.
- Life expectancy less than 12 months.
